# Est-ce que le fumeur connaît les méfaits du tabac?

**DOI:** 10.11604/pamj.2014.19.127.5163

**Published:** 2014-10-03

**Authors:** Hicham Janah, Hanane Elouazzani, Hicham Souhi, Hicham Naji-Amrani, Adil Zegmout, Ismail Abderrahmani Rhorfi, Ahmed Abid

**Affiliations:** 1 Service de Pneumologie, Hôpital Militaire d'Instruction Mohammed V, Rabat, Maroc

**Keywords:** Tabagisme, fumeur, morbidité, smoking, smoker, morbidity

## Abstract

Le tabagisme est une des principales causes de morbidité et de mortalité évitable. Le but de ce travail est d’évaluer les connaissances du fumeur vis-à-vis les risques du tabac et son motivation pour cesser de fumer. Nous avons réalisé une enquête transversale chez les malades tabagiques hospitalisés au service en répondant à un questionnaire précisant leur niveau socio-économique et culturel, l'histoire du tabagisme, les méfaits connus du tabac, la dépendance pharmacologique à la nicotine et leur motivation pour cesser de fumer. Il s'agit de 120 patients, l’âge moyen était de 45 ans±15. Les principaux motifs d'hospitalisation étaient: pathologie tumorale (54%), pathologie infectieuse (35%) BPCO (9%). 85% des patients étaient scolarisés jusqu'au cycle secondaire. Pour l'ensemble des patients, l’âge moyen de la première cigarette était précoce (avant de 20 ans) et la dépendance pharmacologique à la nicotine était légère (82% avaient un score de Fagerström < 8). Plus d'un tiers des patients (38%) avaient au moins fait une tentative d'arrêt. La connaissance des effets néfastes du tabac était variable: les pathologies cancéreuses (88%), les maladies cardiovasculaires (65%) et la BPCO (31%). La majorité des patients (78%) avaient cessé de fumer depuis l'hospitalisation. Notre enquête met en évidence l'intérêt de la médiatisation dans l'information de la population sur les risques du tabac. Les pneumologues, qui prennent en charge la grande majorité de ces patients, doivent donc s'impliquer activement dans ce domaine.

## Introduction

Le tabagisme pose un problème majeur de santé publique car il est responsable d'environ cinq millions de décès par an dont le tiers, soit un million, survient dans les pays en voie de développement. Alors que ses effets nocifs sur la santé sont connus depuis plus d'un demi-siècle, force est de constater que la consommation du tabac ne cesse de croître dans le monde entier. Selon l'Organisation mondiale de la santé, le nombre de décès annuel de 2020 à 2030atteindra dix millions, dont 70% proviendraient des pays en voie de développement.

## Méthodes

Il s'agit d'une enquête transversale type un jour donné réalisée dans le service de pneumo-phtisiologie de l'HMIMV de Rabat. Les patients inclus dans cette étude étaient les sujets fumeurs. En répondant à un questionnaire précisant leur niveau socio-économique et culturel, l'histoire du tabagisme, les méfaits connus du tabac (pathologies cancéreuses, BPCO et maladies cardiovasculaires), la dépendance pharmacologique à la nicotine et leur motivation pour cesser de fumer.

## Résultats

**Figure 1:**
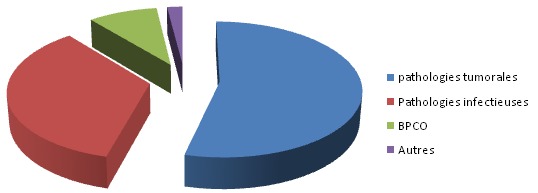
Les principaux motifs d'hospitalisation dominés par les pathologies tumorale et infectieuse

**Figure 2:**
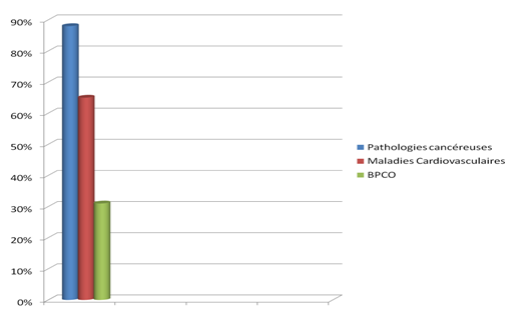
Niveau de connaissance des méfaits du tabac dans notre série

Parmi 310 patients hospitalisés au cours ladite période 120 patients étaient des fumeurs soit une prévalence de 38,7%. Ils étaient tous de sexe masculin. L’âge moyen était de 45 ans±15. Les principaux motifs d'hospitalisation étaient: pathologie tumorale chez 54% des cas, pathologie infectieuse chez 35% des cas, BPCO chez 9% des cas (Figure 1). 85% des patients étaient scolarisés jusqu'au cycle secondaire. Pour l'ensemble des patients, l’âge moyen de la première cigarette était précoce (avant de 20 ans) et la dépendance pharmacologique à la nicotine était légère (82% avaient un score de Fagerström < 8). Plus d'un tiers des patients (38%) avaient au moins fait une tentative d'arrêt. La connaissance des effets néfastes du tabac était variable: les pathologies cancéreuses dans 88% des cas, les maladies cardiovasculaires dans 65% des cas et les BPCO 31% des cas (Figure 2). La majorité des patients (78%) avaient cessé de fumer depuis l'hospitalisation.

## Discussion

Le tabagisme, véritable problème de santé publique, est au centre de l'actualité médicale. Il est le principal facteur de risque des maladies non transmissibles et provoque le décès prématuré de plus de cinq millions de personnes par an à travers le monde, que ce soit par les cancers, les accidents cardiovasculaires, la bronchite chronique ou les bronchites chez les enfants exposés au tabagisme passif. Le Maroc, pays en transition épidémiologique, n'est pas épargné par ce fléau. En effet, les résultats d'une enquête nationale réalisée en 2001 ont montré que parmi les adultes âgés de 20 ans et plus, la proportion de fumeurs est de 34,5% chez les hommes et moins de 1% chez les femmes [1]. Une autre étude menée en collaboration avec l'Organisation mondiale de la santé (OMS), l'United Nations International Children's Emergency Fund (Unicef) et le Centers for Disease Control and Prevention (CDC d'Atlanta), chez les collégiens marocains âgés de 13 à 15 ans en 2001, a montré que 13,5% des élèves ont déjà fumé des cigarettes. Les fumeurs réguliers représentaient 4,5; et 24,3% avaient commencé à fumer avant l’âge de dix ans [2]. D'autres études réalisées au Maroc ont montré que la prévalence du tabagisme varie selon les catégories socioprofessionnelles (milieu scolaire, milieu universitaire, les professionnels de santé, entreprises, administrations) [3, 4].

La prévalence du tabagisme dans notre population est de 38,7%. Ce chiffre est nettement au-dessus de la prévalence nationale estimée par l'OMS à 22,1% [5]. Il touchait majoritairement les hommes. D'une façon générale, le tabagisme féminin est faible. Dans l’étude de l'OMS, on note une prévalence de 1,1 de fumeuse au plan national [5]. Les traditions socioculturelles encore vivaces au Maroc et dans les pays nord africains expliquent cette nette prédominance masculine. De plus, la majorité des fumeurs débutent le tabagisme à un âge précoce avec une moyenne de 19,6 ans dans notre série. Najem et al. [6] dans leur série d'adolescents trouvent que tous les fumeurs ont débuté le tabagisme avant l’âge de 16 ans. Ainsi, il est nécessaire de développer des programmes d'intervention à l'intention de cette tranche d’âge. Le tabac est un produit dangereux, responsable de nombreuses maladies et affectant pratiquement tous les organes [7]. Malgré les dégâts qu'il provoque, sa consommation ne cesse de croître, surtout dans les pays en développement. Malheureusement, ces conséquences sont ignorées par la majorité des sujets interrogés dans notre étude (60,6%). Il faut donc non seulement informer la population sur les dangers du tabagisme mais aussi corriger les croyances erronées. En effet, la lutte contre le tabagisme ne saurait être efficace sans une information suffisante de la population. Cette connaissance était nettement influencée par le niveau d’étude. Il existe une relation statistiquement significative entre la connaissance des conséquences du tabagisme et le niveau d'instruction. Moins on est instruit, moins on est informé des dangers du tabagisme.

## Conclusion

La prévalence du tabagisme reste élevée. Il touche essentiellement les hommes et les adultes jeunes. Malheureusement, les conséquences du tabagisme sont peu connues de la population générale, en dehors du cancer broncho-pulmonaire et des maladies cardiovasculaires. En se référant au niveau d’études, ce sont les sujets les plus instruits qui ont un meilleur niveau de connaissance du tabagisme; ils sont également ceux qui fument le moins. Tous ces résultats doivent réellement inciter à accentuer la sensibilisation sur le tabagisme. Des pays en voie de développement comme le nôtre ne peuvent aucunement faire face aux coûts excessifs engendrés par la prise en charge des maladies liées au tabac. De ce fait, la prévention reste le moyen de lutte le plus adapté.
